# Correlation between the visibility of submandibular fossa and mandibular canal cortication on panoramic radiographs and submandibular fossa depth on CBCT 

**DOI:** 10.4317/medoral.22115

**Published:** 2017-12-24

**Authors:** Seval Bayrak, Husniye Demirturk-Kocasarac, Emre Yaprak, Gulbahar Ustaoglu, Marcel Noujeim

**Affiliations:** 1 Assistant Professor, DDS, PhD, Department of Oral and Maxillofacial Radiology, Abant Izzet Baysal University, Bolu, Turkey; 2 DDS, PhD, Department of Comprehensive Dentistry, The University of Texas Health Science Center San Antonio, Texas, USA; 3 Assistant Professor, DDS, PhD, Department of Periodontology, Kocaeli University, Kocaeli, Turkey; 4 Assistant Professor, DDS, PhD, Department of Periodontology, Abant Izzet Baysal University, Bolu, Turkey; 5 Professor, DDS, MS, Oral and Maxillofacial Radiology Program Director, Department of Comprehensive Dentistry, The University of Texas Health Science Center San Antonio, Texas, USA

## Abstract

**Background:**

To identify a correlation between the submandibular fossa (SF) visibility and mandibular canal (MC) cortication on panoramic image and the depth of SF measured on CBCT and also correlation between the depth of SF and vertical and horizontal location of MC on CBCT.

**Material and Methods:**

500 CBCT scans and panoramic radiographs were evaluated. SF depth types were classified as type I (< 2mm); type II (2-3mm) and type III (> 3mm) on CBCT. Visibility of SF and the cortication of MC on panoramic radiographs were compared with the depth of SF on CBCT. Distances between MC and mandibular inferior, buccal and lingual cortices were measured.

**Results:**

No statistically significant correlation was found between radiolucent appearances of SF, cortication of MC, and depth of SF. The deepest part of the fossa was in the second molar area followed by third and first molars. Negative weak correlations were found between B-MC, L-MC distances and depth of SF.

**Conclusions:**

Visibility of SF and cortication of MC on panoramic radiographs did not correlate with the depth of SF. A marked radiolucent submandibular fossa on panoramic image does not undoubtedly indicate a deep fossa, which emphasizes the importance of 3-D imaging in implant planning.

** Key words:**Cone beam computed tomography, panoramic radiography, dental implants, submandibular fossa, intraoperative complications, mandible, mandibular nerve.

## Introduction

Dental implant therapy is widely applied in clinical practice for the replacement of missing teeth. Accurate determination of anatomical landmarks during the treatment plan is crucial for the clinician to avoid surgical complications. Among these structures, the location of submandibular fossa (SF) and mandibular canal (MC) must be thoroughly evaluated by the clinicians before implant placement in the mandible. Damage to SF by lingual plate perforation during the surgery may lead to severe hemorrhage and subsequent hematoma which have life-threatening consequences due to upper airway obstruction (1,2). In addition, inferior alveolar nerve injury may result in paresthesia or complete anesthesia (3). The probability of damaging these anatomical structures may encourage the clinicians to use unnecessarily short or narrow implants with improper angulations. Thus, a comprehensive preoperative treatment planning including three dimensional radiographic examination is essential for implant placement in appropriate position with optimal length and width to obtain favorable surgical and prosthetic outcomes.

Considerably high levels of radiation exposure is the foremost disadvantage of CT scans. Panoramic imaging has already been routinely used in implant dentistry and it is a fact that many cases actually does not crucially require CT analysis. As a fundamental principle of diagnostic radiology, the patients have to be exposed to as low as reasonably achievable (ALARA) radiation. In a guideline published by European Association for Osseointegration, it has been stated that medical exposure to ionizing radiation must always be justified and results in a net benefit to the patient (4). Accordingly, identifying the cases having high potential for complication during the clinical examination and directing them for CT analysis is the most logical approach to protect patients from unnecessarily additional radiation exposure. Palpation, caliper using or model analysis during the clinical examination phase may provide preliminary information in deciding necessity of CT analysis with respect to the width of alveolar crest. Similarly, panoramic radiographs provides some data about the height of alveolar bone despite image distortion and magnification.

Predicating the presence of a deep SF by panoramic radiographs may provide valuable information which aids the clinicians during the evaluation of the necessity of cone beam computed tomography (CBCT) analysis. Estimating the depth of SF using panoramic radiographs was subject of various previous studies. Presence of diffuse radiolucent appearance at the posterior mandible in panoramic radiographs was referred as the appearance of SF in the previous reports.

Revealing the possible association between the vertical location of MC and the depth of SF may be beneficial for the estimation of the severity of the depth of SF on panoramic radiographs.

Since the alveolar ridge resorption caused by numerous factors such as previous periodontal disease or atrophy due to long-term non-function may affect the accuracy of the results, evaluating the inferior location of MC remains more reasonable for this aim. Besides, the possible correlation between the depth of SF and the appearance of MC and SF on panoramic images may reveal clinical utility of panoramic radiographs at least to warn the practitioner of a potentially deep fossa and the necessity of a 3D imaging modality.

Cone beam computed tomography (CBCT) has been suggested as the golden standard for evaluation of alveolar bone, since it allows three-dimensional analysis of investigated region with high accuracy by providing high-quality images with submillimeter resolution and a low radiation dose (5).

The aim of this study was to detect the presence of a correlation between the SF visibility and mandibular canal cortication on panoramic image and the depth of the fossa measured on CBCT. We also aimed to detect a correlation between the depth of SF and the location of MC in vertical and horizontal directions on CBCT.

## Material and Methods

-Case selection and data acquisition

This study was approved by the Ethics Committee of Abant Izzet Baysal University. A retrospective study was performed using images of 500 patients’ (1000 hemimandibles; 234 males, 266 females; ages between 10 and 87, mean age: 37.49 years) CBCT scans and panoramic radiographs taken for diagnostic assessment of different purposes (i.e. implant surgery, evaluation of the positions of impacted teeth, trauma, temporomandibular joint pathologies, cysts and tumors). CBCT images were acquired using I-CAT 3D Imaging System (Imaging Sciences International, Hatfield, PA, USA) with following parameters: 5 mA, 120 kVp, 16 x 9-12 FOV, and 0.3 mm voxel size. Panoramic radiographs were obtained using Soredex (Cranex Novus, Tuusula, Finland) with 70 kVp and 10 mA.

Patients were categorized by gender and age (group I, ≤20 years (n=252 hemimandible); group II, 21-44 years (n=358 hemimandible); group III, ≥45 years (n=390 hemimandible).

The exclusion criteria included CBCT scans showing congenital and/or developmental disorders, abnormal morphology resulting from trauma and pathologic conditions which potentially affected the area of interest and panoramic radiographs with image deformity and displaying obvious dental pathology affecting the area of interest.

-Measurements and data analysis

**Figure 1:**
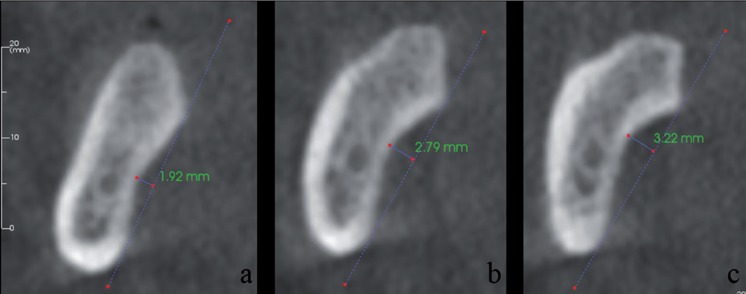
Types of submandibular fossa on cross-sectional CBCT images. Type I, concavity < 2 mm; Type II, concavity = 2 – 3 mm; and Type III, concavity >3 mm.

The deepest part of the SF was determined from cross-sectional images and used for measurements. A line was placed on the most prominent superior and inferior points of the lingual concavity, and a second line was drawn from the deepest point of the concavity perpendicular to the first line. SF types were classified as: Type I, a flat impression < 2 mm deep; Type II, a 2 to 3 mm concavity; and Type III, a concavity > 3 mm (6) (Fig. 1).

**Figure 2:**
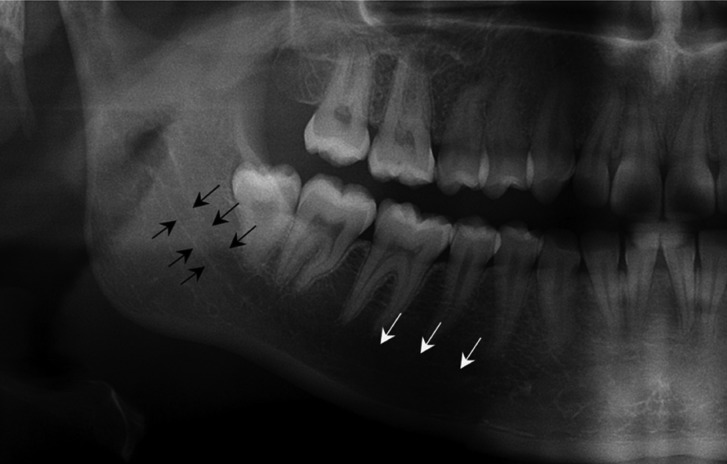
Cropped panoramic radiograph showing mandibular canal cortication (black arrows) and submandibular fossa region (white arrows).

**Figure 3:**
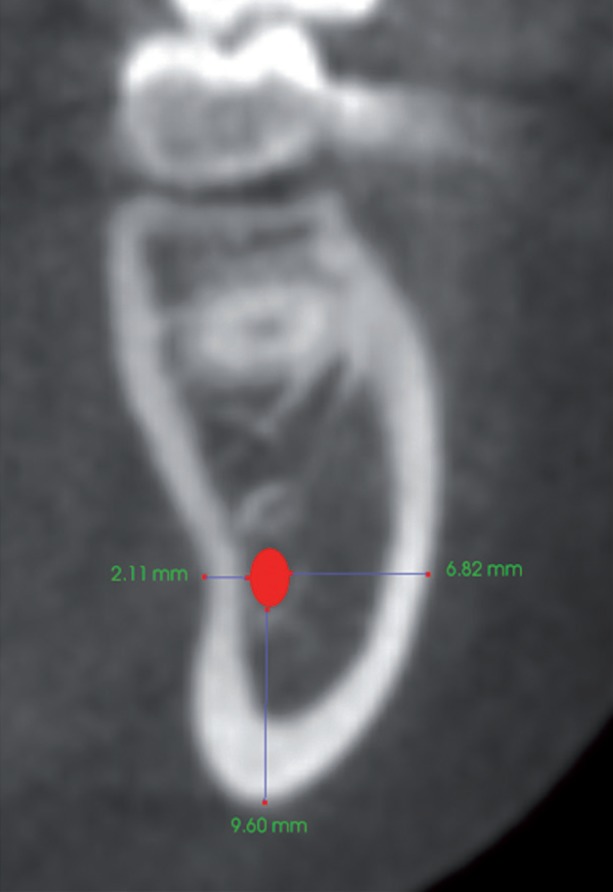
Cross-sectional CBCT image showing bone thicknesses between the mandibular canal and 3 mandibular cortical regions (inferior cortex, buccal cortex, and lingual cortex).

Panoramic interpretations were made based on the presence and absence of an apparent radiolucency indicating the visibility of SF and the cortication of MC in SF region (Fig. 2). These data were then compared with the depth of SF in CBCT by a previously calibrated oral and maxillofacial radiologist who has 7 years of experience. Bone thicknesses between the mandibular canal and 3 mandibular cortical regions (inferior cortex, buccal cortex, and lingual cortex) were measured on cross-sectional images (Fig. 3) as follows:

B-MC: From the buccal cortical border of the mandible to the buccal cortex of the MC 

L-MC: From the lingual cortical border of the mandible to the lingual cortex of the MC

I-MC: From the inferior cortical border of the mandible to the inferior cortex of the MC

All of the measurements were done using I-CAT Vision software (Imaging Science International, Hatfield, PA, USA). After interval of 2 weeks, the measurements in 50 patients were re-evaluated for intra-observer reliability.

-Statistical analysis

All statistical analyses were performed using IBM SPSS for Windows version 20.0 (SPSS, Chicago, IL, USA). Mann-Whitney U test was used to compare sex and age differences and the mean values. The correlation of the depth of the SF with the cortication of the MC and visualization of the SF region, and the depth of the SF with the 3 bone thicknesses (I-MC, L-MC, B-MC) were assessed by Spearman’s correlation test. Kappa test was used to verify the intra-observer reliability. *P* < 0.05 were considered statistically significant.

## Results

Kappa analysis was used to evaluate intra-observer reliability; it ranged between 80% and 90%. A total of 1000 hemimandible from 500 patients (234 males age ranged from 10- 87 years with a mean of 37.68 years and 266 females age ranged from 11- 77 years with a mean of 37.31 years) were evaluated with respect to depth of SF, location of MC, bone thicknesses between the mandibular canal and 3 mandibular cortical surfaces (inferior cortex, buccal cortex, and lingual cortex).

 Table 1 shows descriptive analysis characteristics of the study group. The average SF depth was 2.85 ± 0.8 mm (3.01 ± 0.9 mm for males and 2.9 ± 0.78 mm for females). 55.5 % (555 cases) of the examined SF regions showed flat impressions <2 mm deep (Type I); 37 % (370 cases) had a 2- to 3-mm concavity (Type II), and 7.5 % (75 cases) had a significant concavity >3 mm (Type III) ( Table 2).

**Table 1:**
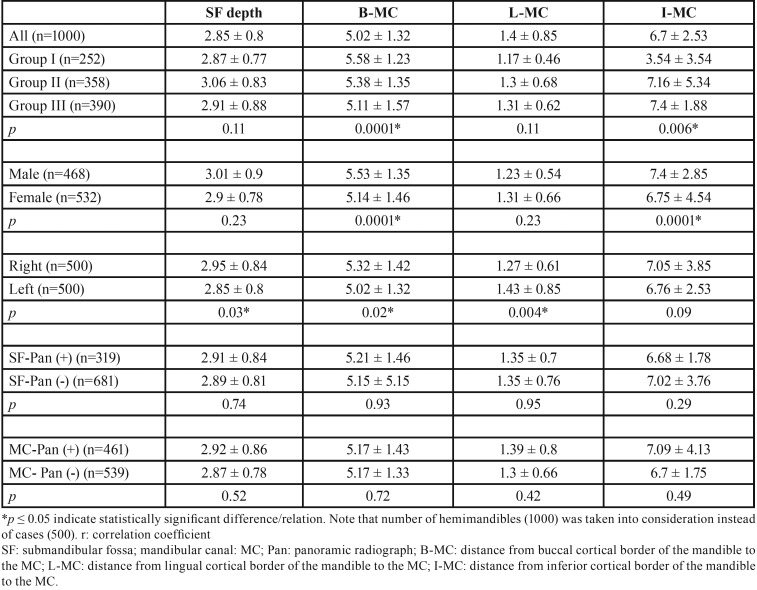
Differences related to age, gender, side and visibility of depth of SF and MC cortication on panoramic radiographs.

The SF region was observed as a radiolucent area in 31.9 % (319 cases) of the hemi-mandibles. An absence of mandibular canal cortication was observed in 46.1 % (461 cases) of the hemi-mandibles ( Table 2).

**Table 2:**
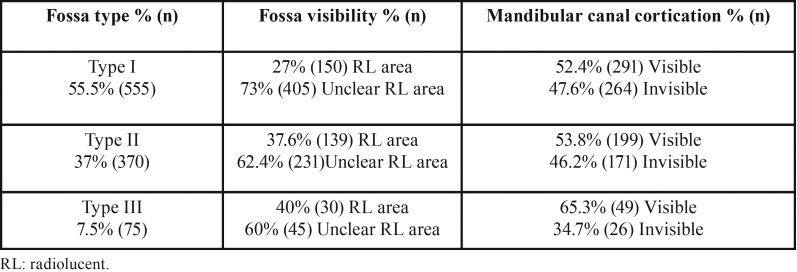
Submandibular fossa visibility and mandibular canal cortication by fossa type.

Differences related to SF depth and MC cortication

The radiolucent appearance of the SF region and MC cortication on panoramic radiographs showed a weak positive correlation (r=0.140 and r= 0.063, respectively) with the type of SF.

No statistically significant correlation was found between radiolucent appearance of SF, cortication of MC, and depth of SF (*p*> 0.05).

However, a weak negative correlation between B-MC, L-MC distance and depth of SF was present (r: -0.12; r: -0.297, respectively). As the SF deepened, decreased thickness of alveolar bone at the buccal and lingual site of the MC was observed. There was no correlation between I-MC and depth of SF ( Table 3). Negative weak correlations were also observed between B-MC, L-MC distance and depth of SF by genders, sides (right and left), visibility of SF and MC cortication on panoramic radiographs. I-MC distance was not significantly correlated with any of these parameters ( Table 3).

**Table 3:**
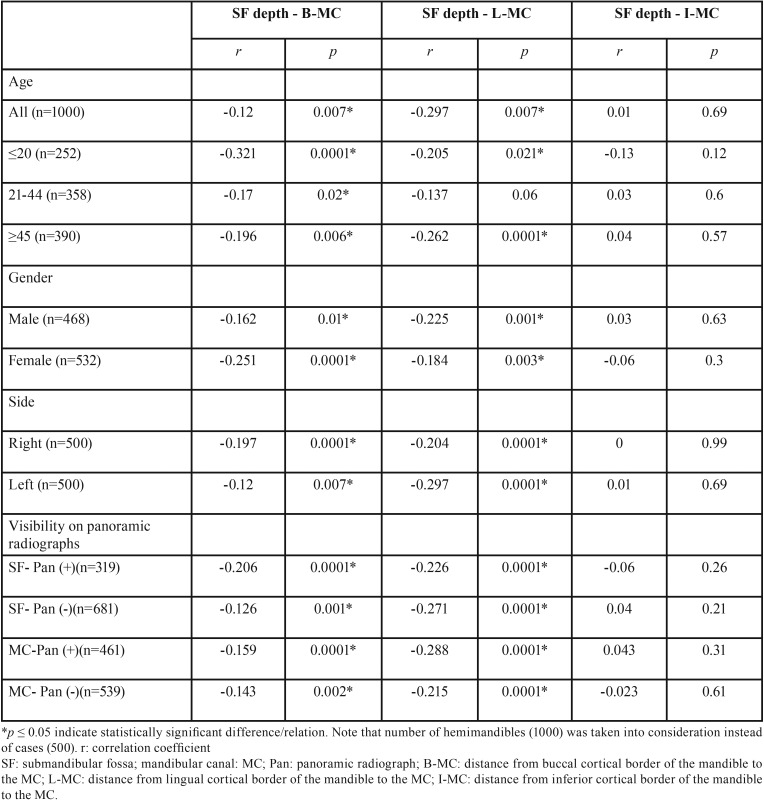
Correlations regarding to age, gender, side and visibility of SF and MC cortication on panoramic radiograph.

The deepest part of the fossa in the right posterior region was in the second molar tooth area (273 out of 500 cases, 54.6 %) followed by third molar (116 cases, 23.2 %), first molar (107 cases, 21.4 %) and second premolar (4 cases, 0.8 %) teeth area. The deepest part of the fossa in the left posterior region was also in the second molar tooth area (309 out of 500 cases, 61.8 %) followed by third molar (123 cases, 24.6 %), first molar (66 cases, 13.4 %) and second premolar (1 case, 0.2%) teeth area.

-Differences related to the age

There was no significant difference in SF depth according to age groups. However, significant relation was seen between I-MC and age groups with an increase in height as the age goes up ( Table 1).

-Differences related to the gender

The average B-MC distance was significantly shorter in females (5.14 ± 1.46 mm) compared with males (5.53 ± 1.35 mm). Similarly, the I-MC distance was shorter in females (6.75 ± 4.54) than in males (7.4 ± 2.85). No significant difference was present between genders regarding to fossa depth and L-MC distance ( Table 1).

-Differences related to side of the mandible

SF depth and B-MC distance was significantly less on the left side (2.85 ± 0.8 mm and 5.02 ± 1.32 mm, respectively) compared to the right side (2.95 ± 0.84 mm and 5.32 ± 1.42 mm, respectively). On the contrary, L-MC distance was shorter on the right side (1.27 ± 0.61 mm) than left side (1.43 ± 0.85 mm). There was no difference on I-MC distance regarding to gender ( Table 1).

## Discussion

Scrutinizing the morphology and dimensions of anatomical landmarks including the SF is important for the prevention of complications during implant placement. The aims of this study were:

• To determine possible association between the visibility of SF and mandibular canal cortication on panoramic radiographs and SF depth on CBCT

• To assess the possible correlations between the depth of SF and the vertical-horizontal locations of the MC

• To investigate the depth of SF in accordance with patient age and sex within a considerably large sample size 

The decreased density of the SF on panoramic image creates a darker background that helped the readers detecting the cortication of the mandibular canal in some cases; however, since the mandibular canal cortication detection varies between modalities and operators, a weak correlation was expected and is not surprising. de Olivera-Santos *et al.* (7), in a CBCT study, analyzed the bone trabeculation in the SF region and reported reduced or invisible trabeculation in most hemimandibles that seemed to effect the cortication of the MC. In assessing the relation between the depth of the submandibular fossa, the visibility of the SF and mandibular canal on panoramic images, we found only a weak positive correlation (r=0.140 and r= 0.063, respectively), a deeper submandibular fossa did not yield the way for better visibility of the mandibular canal and the fossa on panoramic images.

No statistically significant correlation was found between radiolucent appearance as seen on the panoramic image and the depth of SF measured on CBCT. The panoramic image is subject to many sources of distortions due to anatomic variation between patients. The absence of the impression of the fossa on panoramic image might be due to the position of the patient in the machine during panoramic image acquisition. This inconsistency of radiographic image of the SF is most probably also behind the absence of correlation between the radiolucent appearance of SF, cortication of MC and bone thicknesses (B-MC, L-MC and I-MC).

We have found a negative correlation between the SF depth and thickness of the buccal and lingual mandibular bone with respect to the MC. The negative correlation is a normal trend since the increase in SF depth means that the mandible is narrower and the distance from the cortical surfaces to the canal will be reduced.

The depth of the SF reaches its maximum in the area of the second molar on both sides and it decreases in mesial and distal directions to reach the minimum depth in the area of the second premolar. These results are consistent with de Souza *et al.* (5) study findings. Thus we can infer that possibility of perforation of the SF during implant placement in the premolar area is less likely and this risk increases in the distal direction towards to molar area.

The age of the patients did not have any effect on the density of the SF, even though the bone density decreases with age, a difference between the fossa and the surrounding structures will not be detectable.

There was a tendency that the distance between cortical plates and MC was shorter in females than in males. Females have usually smaller stature and softer drawn jaws which explains difference in B-MC and I-MC dimensions between males and females. Even though a difference is also noted between genders in L-MC, the difference was not significant statistically. Clinically, this finding may indicate that potential risk of surgical complication might be more common in females.

Our results revealed that the mandibular canal possibly takes more lingual route in the right mandible than the left. The SF depth and B-MC distance were significantly greater on the right side than the left side; however, the L-MC distance was shorter on the right side than left side (1.43 ± 0.85 mm). On the contrary, Kawashima *et al.* (8) stated that mandibular canal takes more buccal route in the right mandible than the left in both males and females. A similar trend was observed by de Oliveira Junior *et al.* (9) as well, where the mandibular canal seems more buccal on the right side than the left in both genders. The difference may be stem from relatively small sample size of these studies (150 cases and 50 cases, respectively).

We also wanted to investigate the possible correlations between vertical location of MC and depth of SF. An inferior position of the mandibular canal as detected on panoramic image speculates a deeper SF therefore a need for 3-D imaging for better assessment of the crest anatomy and depth of SF. According to the results, there was no correlation between the depth of SF and the vertical location of MC.

We did not find a significant difference in depth of SF regarding to gender and age groups. However, significant relation was present between I-MC and age groups with greater the I-MC height the greater the age. The increase of distance between the inferior cortex of the mandible and the mandibular canal, mainly in the younger age group (group I) is most probably due to the last phase of growth of the body of the mandible in vertical direction. Similarly, B-MC and I-MC differed between genders with males having greater distance comparing to females. In a study conducted by Kawashima *et al.* (8) age and gender did not cause a significant difference between I-MC, B-MC and L-MC with exception of L-MC distance being significantly shorter in males than in females. Authors also stated that B-MC distance was significantly shorter on the right side compared with the left side which is contrary to our study findings. In addition, while L-MC distance was shorter on the right side than left in the present study, Kawashima *et al.* (8) did not report a significant difference. The difference of their and current study results may be due to different number of sample size (155 vs 500 cases, respectively).

In a recent study, de Souza *et al.* (5) evaluated depth of SF among the 100 CBCT scans and observed significant correlation between fossa depth and bone thickness which was measured in a superior location than MC. The authors suggested a greater attention for thick ridges, although favorable, they may be associated with deeper submandibular fossa. In the present study, we found a weak negative correlation between B-MC, L-MC distance and depth of SF. This finding may be explained by the difference in measurement techniques especially for bone thickness.

When a deep undercut is present, the lingual plate may be perforated resulting in hemorrhage during implant surgery, therefore, the angulation of the implant should accommodate the undercut (10). Watanabe *et al.* (11) reported that lingual concavity was present in 36–39 % of their cases. Parnia *et al.* (6) stated that the depth of the SF was more than 2 mm (Type 2 and Type 3 fossa) in 80 % of the study population. In conclusion, the visibility of the SF region and cortication of the MC on panoramic radiographs did not correlate with the depth of the SF. If a correlation could be found we may predict whether the SF is deep or not which may be of a great importance for practitioners who do not have access to 3-D imaging or to avoid extra radiation dose to the patient. However, a marked radiolucent submandibular fossa on a panoramic image does not undoubtedly indicate a deep fossa, which emphasizes the importance of 3-D imaging in implant planning in the mandible.
